# Formation of Porous Structures and Crystalline Phases in Poly(vinylidene fluoride) Membranes Prepared with Nonsolvent-Induced Phase Separation—Roles of Solvent Polarity

**DOI:** 10.3390/polym15051314

**Published:** 2023-03-06

**Authors:** Kuan-Ying Chan, Chia-Ling Li, Da-Ming Wang, Juin-Yih Lai

**Affiliations:** 1 Department of Chemical Engineering, National Taiwan University, Taipei 10617, Taiwan; 2 Material and Chemical Research Laboratories, Industrial Technology Research Institute, Hsinchu County 310401, Taiwan; 3 Graduate Institute of Applied Science and Technology, National Taiwan University of Science and Technology, Taipei 10607, Taiwan

**Keywords:** nonsolvent-induced phase separation, poly(vinylidene fluoride) membranes, crystallization, crystalline phases, water permeability

## Abstract

PVDF membranes were prepared with nonsolvent-induced phase separation, using solvents with various dipole moments, including HMPA, NMP, DMAc and TEP. Both the fraction of the polar crystalline phase and the water permeability of the prepared membrane increased monotonously with an increasing solvent dipole moment. FTIR/ATR analyses were conducted at the surfaces of the cast films during membrane formation to provide information on if the solvents were present as the PVDF crystallized. The results reveal that, with HMPA, NMP or DMAc being used to dissolve PVDF, a solvent with a higher dipole moment resulted in a lower solvent removal rate from the cast film, because the viscosity of the casting solution was higher. The lower solvent removal rate allowed a higher solvent concentration on the surface of the cast film, leading to a more porous surface and longer solvent-governed crystallization. Because of its low polarity, TEP induced non-polar crystals and had a low affinity for water, accounting for the low water permeability and the low fraction of polar crystals with TEP as the solvent. The results provide insight into how the membrane structure on a molecular scale (related to the crystalline phase) and nanoscale (related to water permeability) was related to and influenced by solvent polarity and its removal rate during membrane formation.

## 1. Introduction

**Figure 1:**
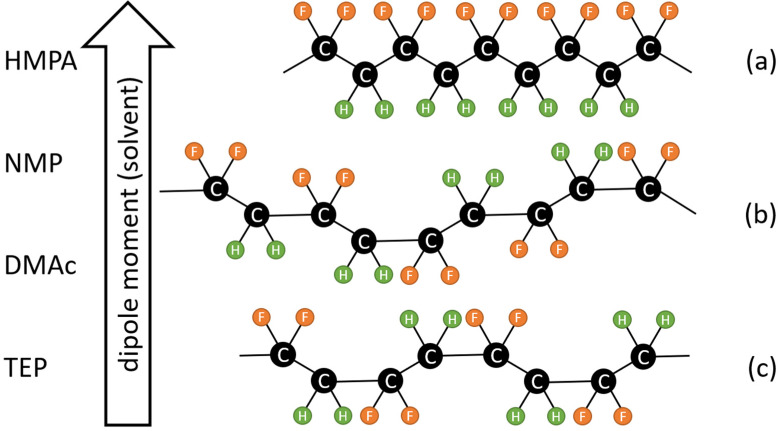
Relationship between PVDF chain conformation and the dipole moment of solvent. PVDF is inclined to form a more polar solution state conformation as it dissolves in a solvent with a higher dipole moment. (**a**) All tans (TTTT); (**b**) TTTG; and (**c**) TGTG′.

Polyvinylidene fluoride (PVDF) is a semi-crystalline polymer and has various chain conformations [1], as depicted in Figure 1. Because of its good thermal stability and chemical resistance, PVDF has been widely used to prepare porous membranes for various applications [2,3,4,5]. Porous PVDF membranes are usually prepared with thermally induced phase separation (TIPS) [4], nonsolvent-induced phase separation (NIPS) [2,6], vapor-induced phase separation (VIPS) [7]), or a combination of them [8,9]. For these phase separation processes, the solubility of PVDF in a casting solution is reduced via either a change in the solution temperature or the introduction of a nonsolvent into the solution to allow the solution to phase separate into polymer-rich (a solution with a high PVDF concentration) and polymer-poor (a solution with a low PVDF concentration) phases. Such phase separation is usually called liquid–liquid (L-L) demixing. Moreover, because of its crystallinity, during membrane formation, PVDF can crystallize from the casting solution, resulting in solid (crystals) and liquid (solution) phases, usually called solid–liquid (S-L) demixing. The competition between L-L and S-L demixing plays an important role in the formation of porous structures and crystalline phases in PVDF membranes [10,11].

**Figure 2:**
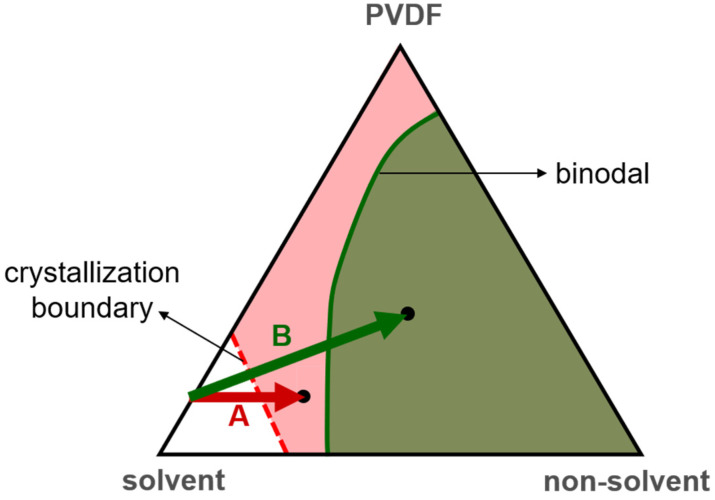
A schematic phase diagram for a PVDF/solvent/nonsolvent ternary solution. PVDF crystallization can occur in the pink and green regions. The green region is called the liquid–liquid demixing region (a region of binodal-type phase separation). Path A schematically represents that the solution composition stays in the crystallization region long enough to allow the polymer to crystallize, and, thus, solid–liquid demixing governs the formation of the membrane structure. Path B represents that the solution composition stays in the crystallization region not long enough before it crosses the binodal, and, thus, liquid–liquid demixing occurs before polymer crystallization and governs the structure formation.

Shown in Figure 2 is a schematic phase diagram for a PVDF/solvent/nonsolvent ternary solution [6,12,13]. Typically, the liquid–liquid demixing region (a region of binodal-type phase separation) is located inside the PVDF crystallization region. During membrane formation via NIPS, the nonsolvent intake into the casting solution changes the solution composition. According to Figure 2, the nonsolvent intake brings the composition of the casting solution first into the crystallization region and then into the liquid–liquid demixing region. However, because the formation of crystalline nuclei takes time, the solution composition needs to stay in the crystallization region long enough to allow for polymer crystallization to occur. On the one hand, if the solution composition stays in the crystallization region long enough to allow the polymer to crystallize and then crosses the binodal, S-L demixing occurs before L-L demixing and governs the formation of the membrane structure, typically resulting in a nodular (spherulitic) structure. On the other hand, if the solution composition stays in the crystallization region not long enough before it crosses the binodal, L-L demixing occurs before polymer crystallization and is the dominant mechanism for structure formation, typically resulting in an asymmetric membrane structure with a dense surface [12]. Therefore, the mass transport of the nonsolvent during NIPS, especially the nonsolvent intake rate, plays a critical role in the competition between S-L and L-L demixing and has a dramatic influence on the structure of the resulting membranes [2,7,10,11,13].

The crystalline phase of PVDF membranes is another interesting property that draws much research attention [3,14]. It has been reported that PVDF crystals have at least four kinds of crystalline phases (α, β, γ and δ) [15,16], with α and β being the major ones [17,18,19]. The α phase, with the polymer chain conformation of trans–gauche (TGTG’), is nonpolar and has high chemical stability. The β phase, with the chain conformation of all trans, is polar and has piezoelectric properties [3,14]. PVDF with piezoelectricity has many interesting applications, such as being used to prepare dense films or porous membranes for flexible pressure or stress sensors [20], self-cleaning filtration [21] and energy harvesting (to transform mechanical energy into electricity) [22]. To develop suitable conditions for the preparation of PVDF membranes with different crystalline phases for various applications, it is essential to generate insight into how crystalline phases are formed during membrane preparation.

The formation of PVDF crystalline phases via melt or solution crystallization has been investigated [3]. During melt crystallization, the α phase has a higher growth rate than other PVDF crystalline phases [23]. Even though it may not nucleate early, the α phase is still the dominant form because it is kinetically favorable [3,23]. Therefore, the α form is the most common crystalline phase obtained from the cooling of the PVDF melt [3,24,25]. For solution crystallization, it has been pointed out that the solvent used to dissolve PVDF plays a dominant role in the formation of crystalline phases because different PVDF chain conformations result in different crystalline phases, and the polymer chain conformation in a solution is strongly influenced by the solvent molecules surrounding the chain [26,27,28]. The effects of the solvent polarity [29,30], solvent evaporation rate [31,32], solvent solubility to PVDF [33,34] and PVDF molecular weight [35] on the formation of crystalline phases have been investigated. Though more investigation is still needed to clarify the detailed mechanism of how the solution state conformation and aggregate structure influence the final PVDF crystalline structure, it is widely accepted that a higher fraction of the polar phase is correlated with a higher polarity solvent [3,29,30]. Nishiyama et al. [29] performed a systematic analysis of the effect of solvent polarity on the formation of crystalline phases by preparing PVDF films with solvents with various dipole moments, using the spin coating process. They confirmed the critical role of solvent polarity in determining the crystalline phases in prepared PVDF films. Using a solvent with a high dipole moment, such as HMPA (hexamethylphosphoramide), PVDF chains were inclined to form a polar conformation (TTTT) and resulted in β-phase crystals. Using a solvent with a lower dipole moment, such as TEP (triethylphosphate), a TGTG’ chain conformation formed, resulting in α-phase crystals. A solvent with a polarity in between HMPA and TEP, such as DMAc (N,N-dimethylacetamide) or NMP (N-methylpyrrolidone), allowed PVDF chains to form in a TTTG conformation, resulting in γ-phase crystals. A schematic presentation of the relationship between solvent polarity and chain conformation is shown in Figure 1. It should be noted that the dominant role of solvent polarity in the formation of crystalline phases is not yet unanimously accepted. Several published papers have stressed the important effects of the solvent power [34] and solvent removal rate [31,32]. More investigations are still needed to clarify the roles of solvent polarity in the formation of PVDF membranes.

In the present work, we prepared PVDF membranes by using various solvents to dissolve PVDF for the preparation of casting solutions, with a focus on the effects of solvent dipole moments on the porous structures, crystalline phases and water permeabilities of the prepared PVDF membranes. To understand how solvent polarity influenced PVDF crystallization during NIPS, analyses using Fourier-transform infrared spectroscopy with attenuated total reflection (FTIR/ATR) were conducted at the surfaces of the cast films at different times during membrane formation to provide information on when crystallization occurred, how the crystalline phases changed during the structure formation, and whether or not the solvents were present in the cast films as the PVDF crystallized. With the FTIR/ATR analyses and the measurements of the viscosities of the casting solutions, we tried to obtain insight into the mechanism responsible for the dependence of membrane permeabilities and crystalline phases on the dipole moments of solvents.

## 2. Materials and Methods

### 2.1. Materials

PVDF Kynar^®^ 760 (MW = 444,000 g·mol^−1^, Arkema) was used to prepare the membranes in the present study. Kynar 760 was dissolved in N,N-dimethylacetamide (DMAc, 99%, Alfa Aesar), N-methylpyrrolidone (NMP, Duksan), triethylphosphate (TEP, ≥99.8%, Sigma-Aldrich, St. Louis, MO, USA) or hexamethylphosphoramide (HMPA, 99%, Sigma-Aldrich) to form the casting solutions for membrane preparation. The properties of the solvents used to dissolve PVDF are listed in Table 1, including the solvents’ dipole moments [36,37,38,39], viscosities [40,41,42,43] and infrared (IR) characteristic peaks [44,45,46,47]. The solvents used are miscible with water which is the nonsolvent used for the preparation of the PVDF membranes. Polyvinylpyrrolidone (PVP) with a molecular weight of 55,000 g·mol^−1^ (from Sigma-Aldrich) was used to characterize the membrane separation performance.

### 2.2. Preparation of PVDF Solutions

PVDF pellets were dissolved in DMAc, TEP, NMP or HMPA at a designated temperature (HMPA, DMAc and NMP: 60 °C; TEP: 110 °C) to form homogenous PVDF casting solutions. It has been reported [7,48] that the PVDF dissolution temperature could influence the porous structures and crystalline phases of PVDF membranes. However, the effects are negligible if the dissolution temperatures are high enough. The dissolution temperatures were selected to allow the temperature effects to be negligible. The PVDF concentrations in the casting solutions were 20 or 25 wt% with HMPA, DMAc or NMP as the solvent and 20 wt% with TEP as the solvent.

### 2.3. Measurement of Viscosities of PVDF Solutions

Solution viscosity was measured by using the Anton Paar MCR 302 rheometer in a 25 mm diameter cone–plate geometry with a 1° cone angle (Model: CP25-1). All tests were performed at 25.0 ± 0.1 °C.

### 2.4. Preparation of PVDF Membranes

The casting solutions were cooled to room temperature for 1 h and then were cast on glass plates at room temperature with a casting knife to form liquid films with a thickness of 300 μm. The cast films were immersed in water (at room temperature) immediately after the casting. After staying in the water bath for 1 day, the formed membranes were air-dried.

### 2.5. Characterization of Membrane Morphologies

The morphologies of the prepared PVDF membranes were examined using scanning electron microscopy (SEM, NovaTM NanoSEM 230, Hitachi Co., Tokyo, Japan). To prepare the samples for the SEM analyses, the PVDF membranes were fractured in liquid nitrogen and then sputter-coated with platinum for 2 min.

### 2.6. Characterization of Crystalline Phases

#### 2.6.1. X-ray Diffraction (XRD)

X-ray diffraction (XRD) was performed to characterize the crystalline phases at the surfaces of the PVDF membranes. The 2θ examined ranged from 10° to 50°, and the scanning rate was 10° per min. The α-crystalline phase of PVDF shows characteristic peaks at 2θ = 18.30°, 19.90° and 26.56° [49]. The β-crystalline phase of PVDF shows characteristic peaks at 2θ = 20.26° and 41.20° [26,49], and the γ-crystalline phase has a peak at 2θ = 20.04° [49]. The characteristic 2θ peaks of the α-, β- and γ-PVDF crystalline phases are summarized in Table 2.

#### 2.6.2. Infrared (IR) Spectroscopy

Different PVDF crystalline phases have different polymer chain conformations, thus having different characteristic peaks in the IR absorption spectra [3]. We conducted FTIR/ATR analyses with an IR spectrometer (Perkin Elmer Spectrum 100) to characterize the crystalline phases at the surfaces of the PVDF membranes. The α-crystalline phase can be characterized by the absorption peaks at 760 and 975 cm^−1^ [50,51], the β phase by the peaks at 840 and 1275 cm^−1^ [50,51] and the γ phase by the peaks at 834 and 1234 cm^−1^ [50,51]. The IR absorption characteristic peaks for the three kinds of PVDF crystalline phases are summarized in Table 2.

For PVDF membranes with only α- and β-crystalline phases, it was reported in the literature [19,52] that the mass fraction of the α phase (F_α_) in PVDF crystals can be calculated from the absorbance of the peaks at 760 cm^−1^ and 840 cm^−1^:

F_α_ = A_α_/[A_α_ + (K_α_/K_β_) × A_β_] (1)

where A_α_ and A_β_ are the absorbances at 760 cm^−1^ and 840 cm^−1^, respectively, and K_α_ (6.1 × 10^4^ cm^2^/mol) and K_β_ (7.7 × 10^4^ cm^2^/mol) are the corresponding absorption coefficients. The fraction of β-phase crystals (F_β_) can be obtained using the expression F_β_ = 1 − F_α_.

### 2.7. Characterization of the Evolution of Crystalline Phases during Membrane Formation

**Figure 3:**
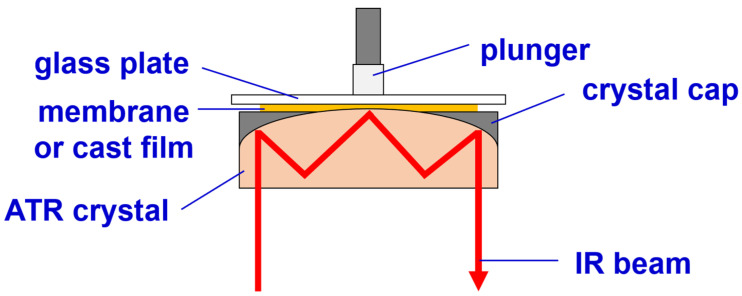
The setup for performing FTIR/ATR analysis at the surface of a membrane or a cast film.

FTIR/ATR analyses with a reflection mode were conducted at the surfaces of the cast films during membrane formation to provide information on when crystallization occurred and how the crystalline phases changed during the formation of the surface structures. The PVDF solution was cast on a glass plate with a casting knife to form a liquid film with a thickness of 300 μm. The glass plate with the liquid film was then turned upside down and placed on top of an ATR crystal (ZnSe), as shown in Figure 3. An IR beam was passed through the ATR crystal and reflected off the crystal surface in contact with the liquid film. The duration of a single IR spectrum measurement is about one minute. With such a setup, FTIR spectroscopy analysis can be performed at the surface of the liquid film. Note that the liquid film might have been squeezed out from the gap between the ATR crystal and the glass plate because of the gravitational force of the glass plate and plunger (see Figure 3) if the glass plate was too heavy or the polymer concentration in the liquid film was too low. We observed that the liquid film would have a viscoelasticity high enough to support the glass plate we used and would not be squeezed out from the gap if the polymer concentration was high enough. To conduct IR spectroscopy on the surface of the cast films during membrane formation, the cast films were placed in contact with the ATR crystal, as discussed above, after they were exposed to water vapor or immersed in water at different times. Typically, the analyses were performed at the following time points during membrane formation: immediately after the solution casting, 5 min after the cast film was immersed in water, and after the membrane was formed.

With the IR spectra obtained with the FTIR/ATR analyses at different times during membrane formation, information on when PVDF crystals formed and how the crystalline phases evolved was obtained by analyzing the changes in the absorbance of the IR characteristic peaks of the α-, β- and γ-crystalline phases (listed in Table 2). With the FTIR/ATR analyses, we also examined the change in the solvent content at the film surfaces during membrane formation by analyzing the changes in the absorbance of the IR characteristic peaks of HMPA, NMP, DMAc and TEP. The characteristic IR peaks of the four solvents are listed in Table 1.

### 2.8. Measurement of Degrees of Crystallinity of Membranes

Differential scanning calorimetry (DSC, TA Q25) was used to measure the degrees of crystallinity of the PVDF membranes. Approximately 5 mg of each of the PVDF membrane samples was sealed in an aluminum alloy pan and heated from 50 to 200 °C with a heating rate of 10 °C/min under a constant flow of nitrogen gas. The crystallinity of each PVDF membrane (X_c_) was calculated via the expression X_c_ = ΔH_f_/${\Delta H}_{f}^{0}$, where ${\Delta H}_{f}^{0}$ = 105 J/g [53] is the crystallization heat per gram of ideal PVDF crystals, and ΔH_f_ is the crystallization heat per gram of the PVDF samples measured with DSC.

### 2.9. Characterization of Water Permeabilities and PVP Retentions of Membranes

Membrane samples with an effective area of 13.4 cm^2^ were used for the measurement of the water permeabilities of the membranes. The transmembrane pressures were controlled at 1 or 3 bars. The membrane permeability to water was calculated using the following equations:

J = V/(A × t)(2)

P = J/ΔP(3)

where J is the water flux (L/(m^2^∙h), LMH); V the permeate volume (L); A the effective membrane surface area (m^2^); t the filtration time (h); P the permeability of the membrane (L/(m^2^∙h∙bar), LMH/bar); and ΔP the transmembrane pressure (bar). Note that the change in water permeability can only reflect the effects on connected pores, not those on closed pores.

A feed solution of 50 ppm PVP (MW = 55,000 g·mol^−1^) was used to characterize the separation performance of the prepared PVDF membranes. An ultraviolet/visible spectrophotometer (UV/Vis, CARY 300 nc, Agilent Technologies, Santa Clara, CA, USA) was used to determine the PVP concentrations in the feed and permeate, with the absorbance at 193 nm. The retention of PVP was calculated using the following equation:

R = (1 − C_p_/C_f_) × 100% (4)

where R is the retention of PVP; C_p_ the PVP concentration in the permeate; and C_f_ the PVP concentration in the feed.

## 3. Results and Discussion

### 3.1. Crystalline Phases of PVDF Membranes Prepared with NIPS—Crystallization in Solvent-Rich and Solvent-Poor Environments

**Figure 4:**
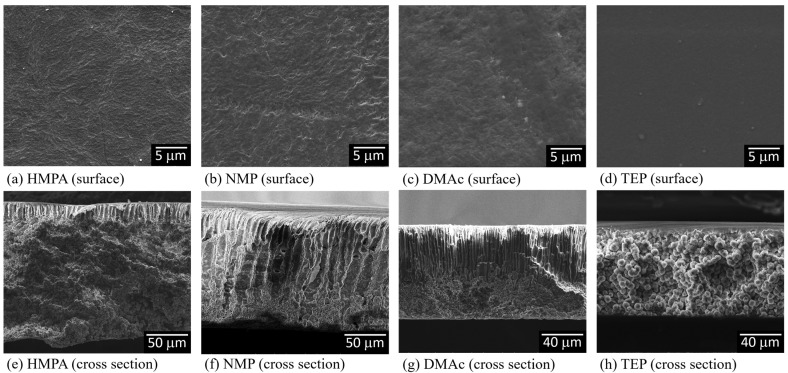
SEM images of the surfaces and cross-sections of PVDF membranes prepared with nonsolvent-induced phase separation, with HMPA, NMP, DMAc or TEP used as the solvent to dissolve PVDF.

**Figure A1:**
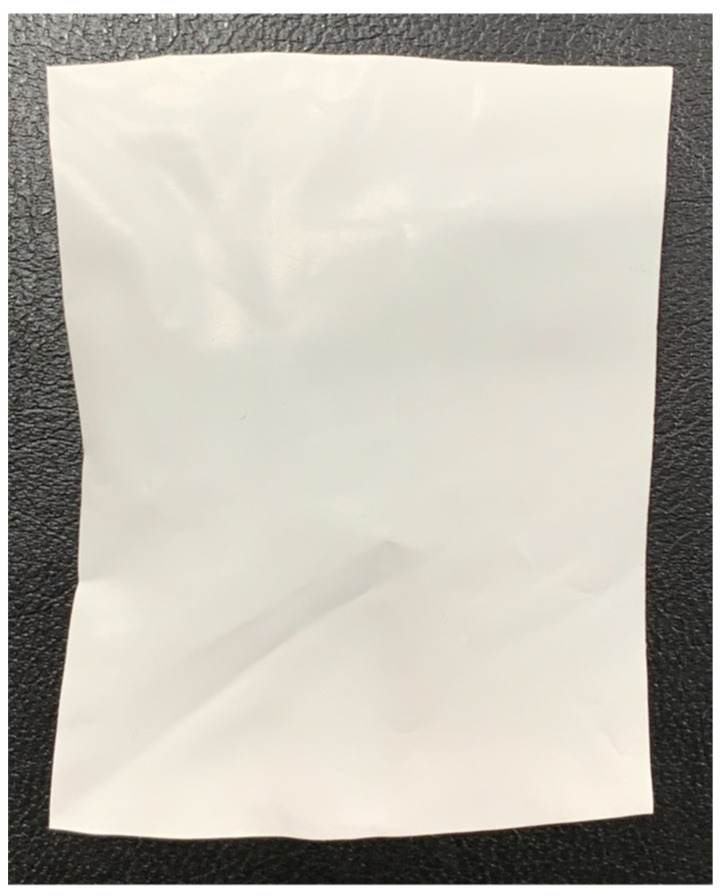
The photo of a PVDF membrane prepared by immersing the PVDF/NMP (20 wt% of PVDF) solution in water.

We prepared PVDF membranes with nonsolvent-induced phase separation (NIPS). After solution casting, the cast films were immediately immersed in a water bath. All the formed membranes look similar to the photo presented in Figure A1 (Appendix A). The membrane structures were examined with SEM, and the images of the membrane surfaces and cross-sections are shown in Figure 4. The obtained structures were all asymmetric with dense surfaces, the typical structure formed via L-L (liquid–liquid) demixing [12,54]. The ternary phase diagrams of PVDF-DMAc-water, PVDF-NMP-water, PVDF-HMPA-water and PVDF-TEP-water have been investigated [12,55,56], and the results show that for all four systems, the L-L demixing regions were inside the polymer crystallization regions, similar to the schematic phase diagram shown in Figure 2. The results indicate that, with NIPS, during membrane formation, the nonsolvent (water) intake into the four casting solutions was too fast to bring about S-L demixing before the onset of L-L demixing. In other words, L-L demixing preceded S-L demixing and dominated the structure formation.

**Figure 5:**
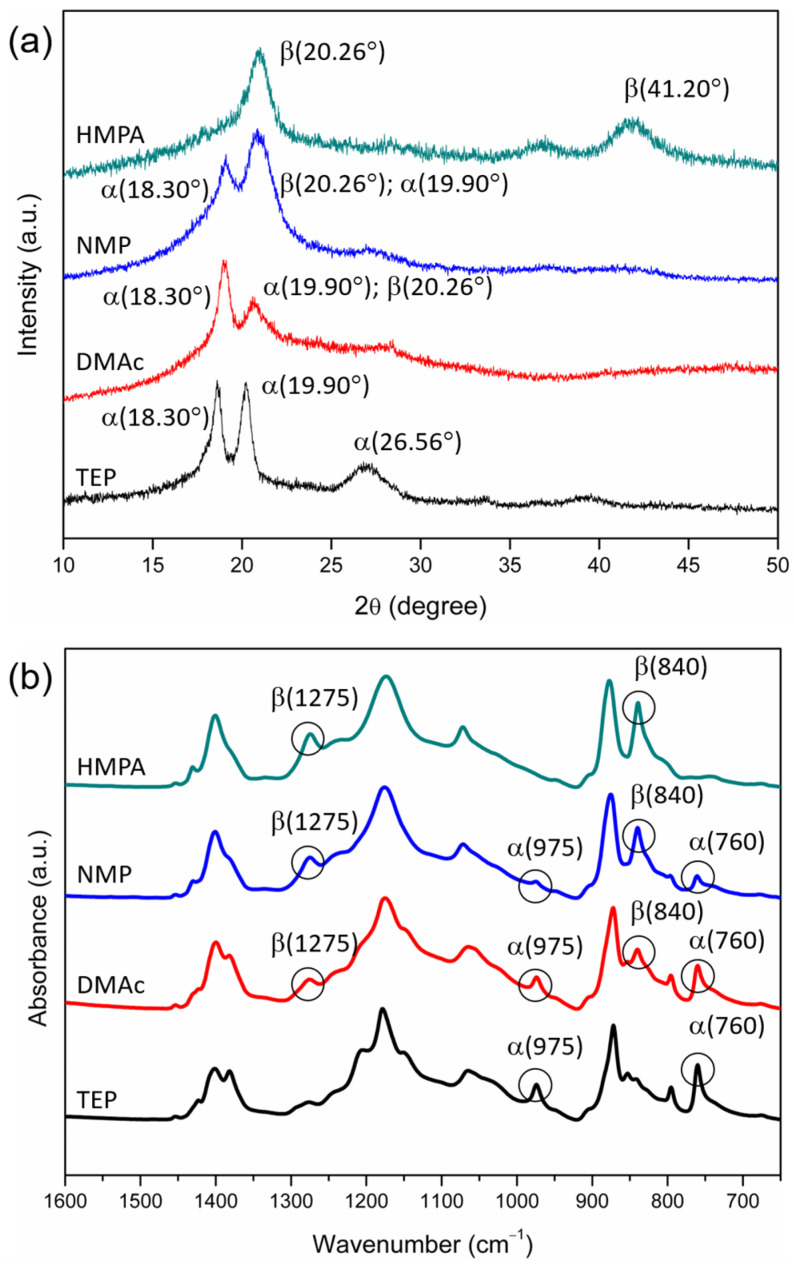
XRD (**a**) and FTIR/ATR (**b**) spectra of the surfaces of PVDF membranes prepared with nonsolvent-induced phase separation, with HMPA, NMP, DMAc or TEP used as the solvent to dissolve PVDF.

XRD and FTIR/ATR analyses were performed to examine the crystalline phases at the membrane surfaces. The results are shown in Figure 5. The surfaces of the resulting membranes were all dense, but their crystalline phases were different. The XRD spectra reveal that membranes prepared with TEP as the solvent contained α-phase crystals (with 2θ peaks at 18.30° and 19.90°), and those prepared with HMPA did not have α-phase crystals (with no characteristic peak at 18.30°) but contained β or γ crystals (with a peak around 20°). The membranes prepared with NMP or DMAc as the solvent contained both nonpolar (α) and polar (β or γ) crystals. The FTIR spectra verified that membranes prepared with TEP as the solvent contained only α crystals (with peaks at 760 cm^−1^ and 975 cm^−1^) and had no β or γ crystals. With HMPA as the solvent, the membrane surface mainly contained β crystals (with characteristic peaks at 840 cm^−1^ and 1275 cm^−1^). With NMP or DMAc as the solvent, the crystalline phase was a mixture of α and β crystals.

As revealed in the asymmetric membrane structures shown in Figure 4, for the NIPS process, L-L demixing preceded S-L demixing. Under this circumstance, PVDF crystallization occurred in the polymer-rich domains formed after L-L demixing. The solvent content in the polymer-rich domains decreased during membrane formation because the solvent contained was continuously extracted out by the water. If the PVDF had crystallized in the initial period after L-L demixing, the solvent content in the crystallization environment might still have been high. However, crystallization would have occurred in an environment with a low solvent content if the PVDF crystallized after much of the solvent contained in the polymer-rich domain had been removed. In other words, PVDF might be crystallized in “solvent-rich” and “solvent-poor” environments during membrane formation. The lapses of time of the solvent-rich and solvent-poor periods were strongly dependent on the solvent removal rate in the coagulation bath.

**Figure 6:**
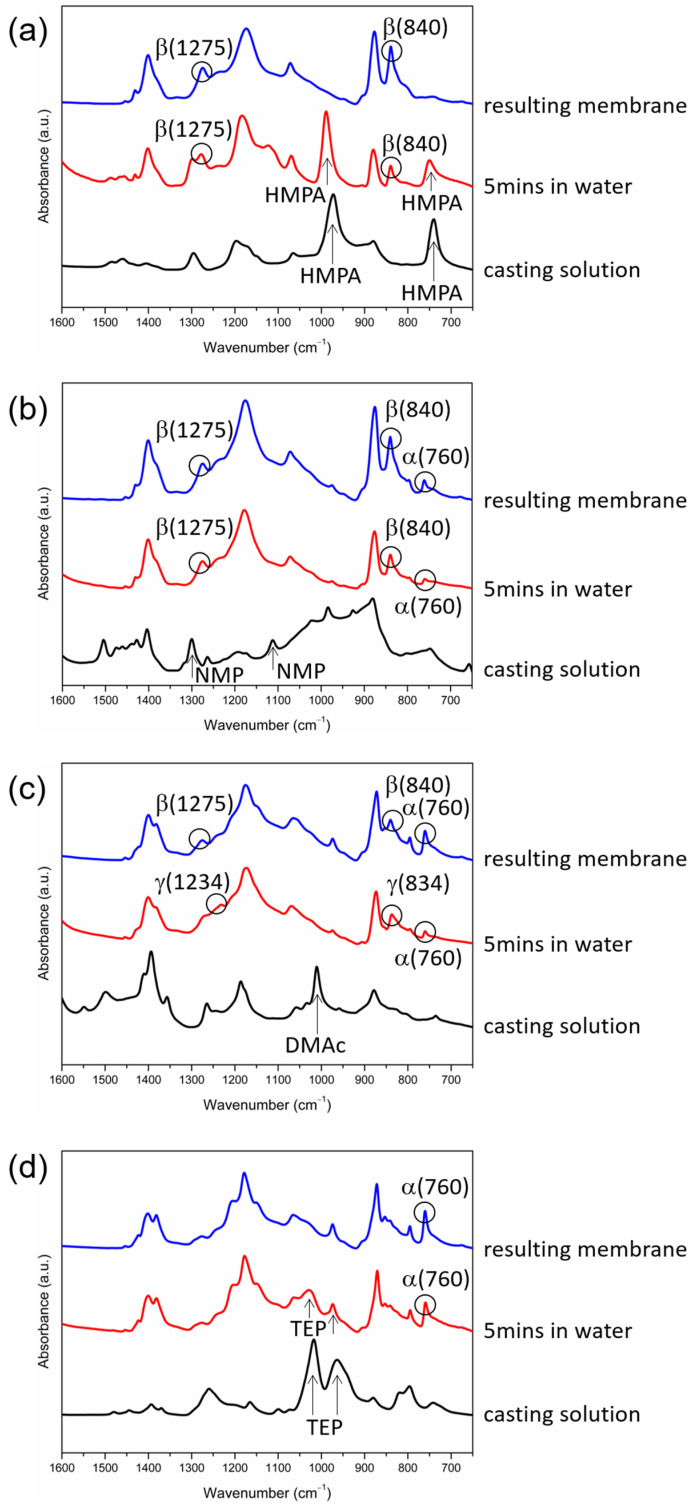
FTIR/ATR spectra of the surfaces of PVDF cast films at three time points during membrane formation (immediately after the solution casting, after 5 min in water, and after the membranes were formed) with HMPA (**a**), NMP (**b**), DMAc (**c**) or TEP (**d**) as the solvent.

To obtain insight into how the solvent removal rate affected the formation of the crystalline phases, FTIR/ATR analyses were conducted at the surfaces of the cast films during membrane formation to provide information on when PVDF crystallization occurred and if a solvent was present in the crystallization environment. The analyses were performed at three time points during membrane formation (immediately after the solution casting, 5 min after the immersion of the casting solutions in water, and after the membranes were formed). The results are shown in Figure 6.

The IR peaks corresponding to HMPA (744 cm^−1^ and 992 cm^−1^) can be clearly seen after the immersion of the cast film in water for 5 min (Figure 6a), indicating that HMPA still existed at the film surface at that moment. During the 5 min in water, the PVDF at the film surface crystallized, evidenced by the occurrence of the two characteristic IR peaks corresponding to the β-PVDF crystalline phase (840 cm^−1^ and 1275 cm^−1^). The results suggest that the removal rate of HMPA in water was low, still allowing enough HMPA to stay in the cast film during the 5 min in water and providing a high-polarity environment for the polar β-crystalline phase to form. Similar results were obtained with TEP as the solvent. TEP was present in the cast film (with IR peaks at 966 cm^−1^ and 1021 cm^−1^) after the film was immersed in water for 5 min, during which the PVDF crystallized to form the α-crystalline phase (with IR peaks at 760 cm^−1^ or 975 cm^−1^), as indicated in Figure 6d. Similar to the case with HMPA as the solvent, the removal rate of TEP in water was low, allowing enough TEP to stay in the polymer-rich domains to provide an environment for the α-PVDF crystalline phase to form. From the results, we can deduce that as PVDF crystallized in the coagulation bath, solvent polarity dominated the formation of crystalline phases if the solvent removal rate from the cast film was low enough to allow a sufficient amount of solvent to be present in the crystallization environment to influence PVDF crystallization. In other words, with HMPA or TEP as the PVDF solvent, the solvent-rich periods for crystallization could last for at least 5 min.

For the case with NMP or DMAc as the solvent, the IR characteristic peaks corresponding to the solvent (1112 cm^−1^ and 1300 cm^−1^ for NMP, and 1011 cm^−1^ for DMAc) disappeared after the cast film was immersed in water for 5 min (Figure 6b,c), indicating that the NMP and DMAc removal rates from the cast film were higher compared with those of HMPA and TEP, and NMP and DMAc existed in the film for less than 5 min. During the 5 min period in water, PVDF crystallized in the polymer-rich domains and formed β- and α-crystalline phases when NMP was the solvent and γ- and α-crystalline phases when DMAc was the solvent. A possible mechanism for this is that, after L-L demixing, the γ phase formed in the initial period when the DMAc concentration in the polymer-rich domains was still high enough (the solvent-rich period), and the α phase formed in the later period when the DMAc content was too low to induce γ-phase crystals (the solvent-poor period). For the case with NMP as the solvent, the mechanism was similar: the β phase formed in the solvent-rich period, and the α phase formed in the solvent-poor period. By comparing the value of the characteristic IR peak of the α-phase (760 cm^−1^) at the surface of the cast film after 5 min in water with that at the surface of the resulting membrane (Figure 6b,c), it is known that the α-PVDF crystalline phase continued to form in water after 5 min of immersion and during the drying stage to form the membrane. The results show that even without the solvent present in the cast film, the α-PVDF crystalline phase still formed. Without the solvent present, PVDF chains were mainly surrounded by other polymer chains, so the crystallization environment was similar to that of melt crystallization. For the melt crystallization of PVDF, the dominant crystalline phase was α because it was kinetically favorable [3]. Our results also show that PVDF was inclined to form the kinetically favored α phase in the solvent-poor crystallization environment.

The above discussion reveals the important role of the solvent content in the crystallization environment in the formation of crystalline phases. On the one hand, as PVDF crystallized in the solvent-rich environment, the solvent concentration was high enough to govern the formation of crystalline phases. On the other hand, as crystallization occurred in the solvent-poor environment, the solvent concentration was too low to influence PVDF crystallization, and the formed crystalline phase was the kinetically favored α phase. With HMPA as the solvent, only the solvent-governed β-crystalline phase was observed, indicating that PVDF crystallized mainly in the solvent-rich environment. With NMP or DMAc as the solvent, both the solvent-governed phase and the kinetically favored α phase were obtained, indicating that PVDF crystallized in both solvent-rich and solvent-poor environments. With TEP as the solvent, both the solvent-governed and kinetically favored phases were α. The IR characteristic peak of the α phase (760 cm^−1^) occurred in the first 5 min in water (the solvent-rich period) and continued to grow during the drying stage to form membranes (the solvent-poor period), as shown in Figure 6d, confirming that the α-crystalline phase formed in both solvent-rich and solvent-poor environments.

**Figure 7:**
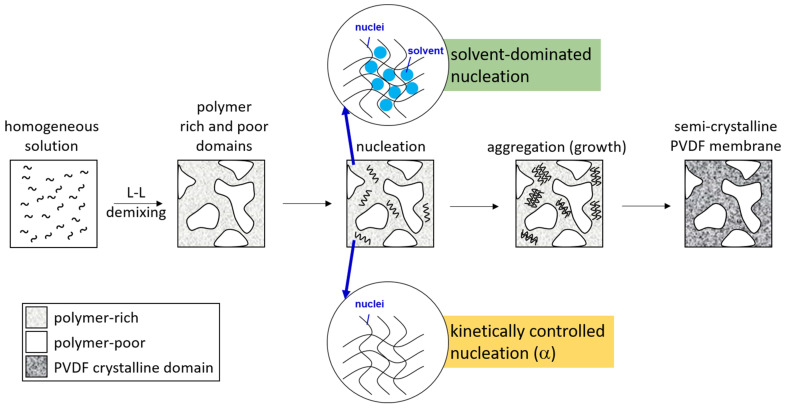
Schematic presentation of the formation mechanism of PVDF membranes.

Presented in Figure 7 is a schematic description to summarize the membrane formation mechanism discussed above. With NIPS, L-L demixing preceded S-L demixing and dominated the structure formation. PVDF crystallization then occurred in the polymer-rich domains to form semi-crystallized membranes. As PVDF crystallized in the “solvent-rich” environment, solvent polarity governed the formation of crystalline nuclei and dominated the formation of crystalline phases. As PVDF crystallized in the “solvent-poor” environment, kinetically favorable nuclei formed and the α-crystalline phase dominated. For systems with low solvent removal rates (with HMPA or TEP as the solvent), PVDF mainly crystallized in the “solvent-rich” environment, and for systems with high solvent removal rates (with NMP or DMAc as the solvent), PVDF crystallized in both “solvent-rich” and “solvent-poor” environments, and both the polar crystalline phase (solvent-dominated) and nonpolar crystalline phase (kinetically favorable) formed.

It can also be observed in Figure 6c that the peaks at 834 cm^−1^ and 1234 cm^−1^ (corresponding to the γ phase), occurring after the immersion of the cast film in water for 5 min, shifted to 840 cm^−1^ and 1275 cm^−1^ (corresponding to the β phase) after the membrane was formed. It has been reported that γ-PVDF crystals could transform into β crystals when they were subject to mechanical stress [57]. Such a mechanism may explain the shift of the characteristic IR peaks we observed since the polymer in the polymer-rich domains near the surface of the cast film was subject to capillary stress induced by the evaporation of the liquid contained in the polymer-poor domains during the drying stage [58]. We propose here that the γ-crystalline phase formed in water can transform into the β phase when subjected to the capillary stresses induced by liquid evaporation during the drying stage. More investigations are still needed to verify the proposed mechanism.

### 3.2. Effects of Solvent Dipole Moments on Formation of Crystalline Phases for PVDF Membranes Prepared with NIPS

**Figure 8:**
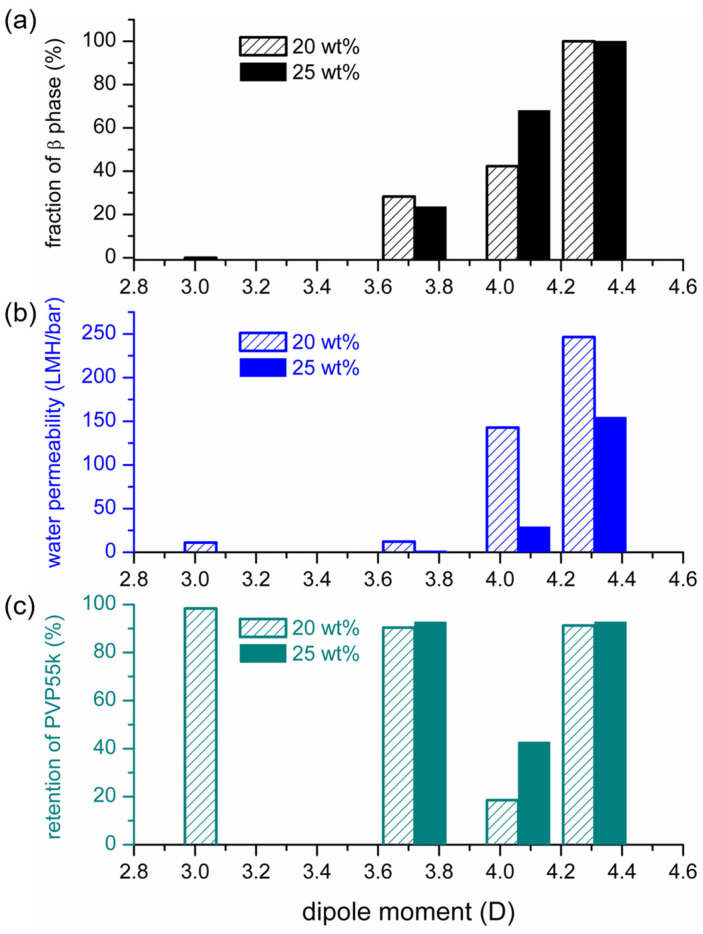
Dependence of the fractions of β-crystalline phase (**a**), water permeabilities (**b**) and retention of PVP (**c**) of PVDF membranes on the dipole moments of the solvents used to dissolve PVDF.

The results shown in Figure 5 indicate that the PVDF membranes prepared with NIPS mainly contained α-phase and β-phase crystals, and the ratio of the two crystalline phases depended on the polarities of the solvents used to dissolve PVDF. To quantitatively describe the dependence, we characterized the mass fraction of the β phase (F_β_) in PVDF crystals by using the procedures described in §2.6.2. The relationship between F_β_ and the solvent dipole moment is depicted in Figure 8a. It can be seen that a higher F_β_ was obtained when the solvent with a higher dipole moment was adopted to dissolve PVDF. Furthermore, similar trends were obtained for 2 PVDF concentrations in the casting solutions (20 and 25 wt%). The results for the fraction of the β phase and the degrees of crystallinity in the PVDF membranes prepared in the present work are listed in Table 3. Note that the monotonous increase in F_β_ with an increasing solvent dipole moment may seem to not be consistent with the results reported in the work of Tao et al. [34], which stressed the importance of solvent power instead of solvent polarity. Such discrepancy could result from the effect of the dissolution temperature. It was identified in [7,48] that the PVDF dissolution temperature could influence the crystalline phase of prepared PVDF membranes, and the effect could only be negligible if the dissolution temperatures were high enough. The dissolution temperatures used in the present work were selected to allow the temperature effects to be negligible (HMPA, DMAc and NMP: 60 °C; TEP: 110 °C). The dissolution temperature used in the work of Tao et al. [34] was 80 °C with HMPA, DMF, TEP or TMP as the solvent. The dissolution temperature of 80 °C should be high enough with HMPA and DMF but not high enough with TEP or TMP as the solvent. The effect of the dissolution temperature on the formation of crystalline phases with TMP as the solvent has actually been pointed out in their work [34]. We believe the role of solvent polarity can be identified more clearly without the interference of the effect of dissolution temperature.

**Figure 9:**
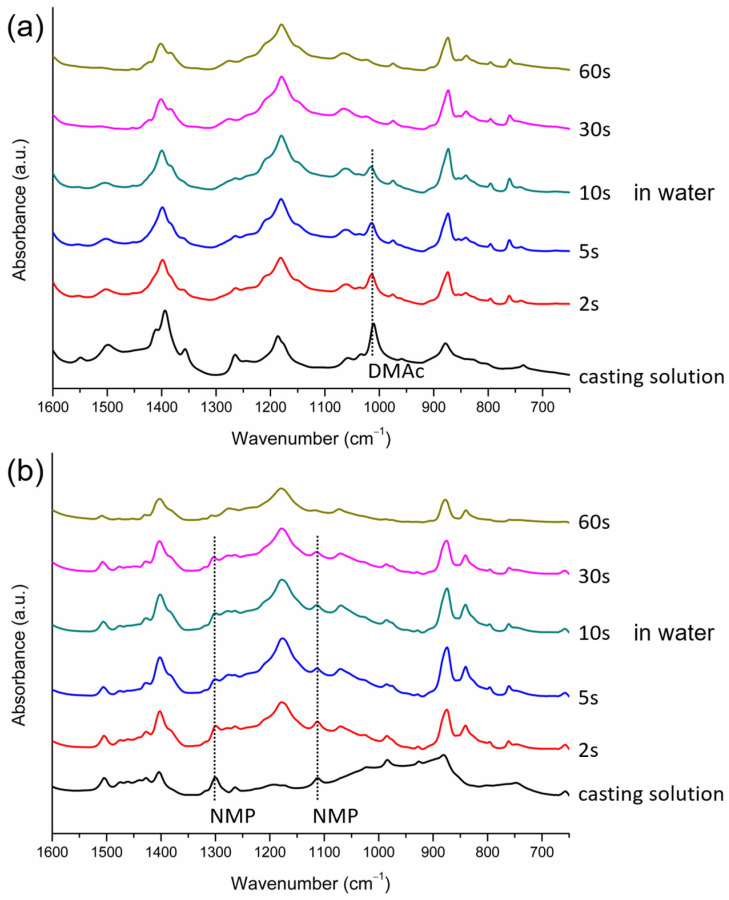
FTIR/ATR spectra of the surfaces of PVDF cast films at different times after immersion in water, with DMAc (**a**) or NMP (**b**) as the solvent.

**Figure 10:**
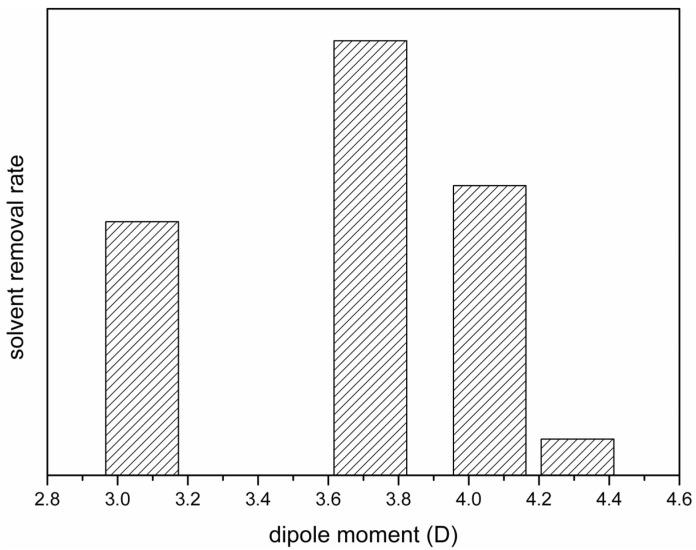
Schematic presentation of the dependence of the solvent removal rate in coagulation bath on its dipole moment.

We also investigated the solvent removal rate of the cast film in the coagulation bath because it determined the lapses of time for the solvent-rich and solvent-poor periods and played an important role in the formation of crystalline phases, as discussed in the preceding section. The data presented in Figure 6a show that the removal rate of HMPA from the surface of the cast film was low and that the content of HMPA still remained high 5 min after the film was immersed in water. The removal rates of NMP and DMAc from the surface of the cast film were higher compared with that of HMPA, and almost no NMP or DMAc remained at the surface after 5 min of immersion, as indicated in Figure 6b,c. The removal rates of NMP and DMAc could not be differentiated from the IR spectra obtained after the immersion of the cast films in water for 5 min. Therefore, FTIR/ATR analyses were conducted at the surfaces of the cast films at shorter time points after the films were immersed in water. The results are presented in Figure 9. It can be seen that the characteristic peak corresponding to DMAc could not be detected after immersion for 30 s and that the peaks corresponding to NMP disappeared after immersion for 60 s. The results indicate that during membrane formation, DMAc was almost completely removed from the surface of the cast film after 10–30 s in water and that NMP was removed after 30–60 s in water, revealing that the removal rate of DMAc was higher than that of NMP. The characteristic peaks corresponding to TEP were detected at the surface of the cast film after 5 min in water, as indicated in Figure 6d. However, the peaks were not as high as those in the casting solution (before immersion in water). Note that, as seen in Figure 6a, the peaks corresponding to HMPA had similar heights before and after the film was immersed in water. Since TEP still existed at the film surface after 5 min in water, the removal rate of TEP was lower than those of DMAc and NMP. The lower height of the peaks for TEP after 5 min in water indicates that TEP has a higher removal rate than HMPA. On the basis of the FTIR/ATR analyses discussed above, we could determine the relative removal rates of HMPA, NMP, DMAc and TEP in the coagulation bath, though the exact removal rates were still not known. Shown in Figure 10 is a schematic presentation of the dependence of the solvent removal rate on its dipole moment.

With the relative solvent removal rates presented in Figure 10, we can satisfactorily explain the dependence of F_β_ on the solvent dipole moments shown in Figure 8. With HMPA, NMP or DMAc as the solvent, the solvent removal rate decreased with the increasing solvent dipole moment. A lower solvent removal rate allowed the solvent to stay longer at the surface of the cast film, giving a longer solvent-rich period for solvent-governed crystallization to occur. Since the three solvents induced the formation of the polar crystalline phase, longer solvent-rich periods resulted in a higher ratio of the polar crystalline phase. With TEP as the solvent, only the nonpolar α-crystalline phase formed for both solvent-rich (solvent-governed) and solvent-poor (kinetically favored) crystallization. Therefore, with TEP as the PVDF solvent, F_β_ was the lowest, even though the solvent removal rate was not the highest.

On the basis of the above discussion, it can be summarized that the monotonous increase in F_β_ with the increasing solvent dipole moment can be reasoned by considering the solvent removal rate of the cast film in the coagulation bath and the inducement of the α-crystalline phase with a solvent with a low dipole moment or an environment with a low solvent concentration.

### 3.3. Effects of Solvent Dipole Moments on Water Permeabilities of PVDF Membranes Prepared with NIPS

We also measured the water permeabilities through the PVDF membranes prepared with NIPS, using solvents with various dipole moments. The results are shown in Table 3 and Figure 8b. It can be seen that the water permeability increased monotonously with the increasing solvent dipole moment. As the PVDF concentration in the casting solutions increased from 20 to 25 wt%, the water permeability decreased, but the trend of its dependence on solvent polarity was still similar.

The high water permeability of the PVDF membrane prepared with HMPA as the solvent has also been reported in the work of Bottino et al. [12]. The authors proposed that the high permeability stemmed from the low polymer concentration at the surface of the cast film as phase separation occurred. The low polymer concentration resulted in a more porous membrane surface, explaining why the membrane permeability was high. The data presented in Figure 6a show that during membrane formation, the removal rate of HMPA from the surface of the cast film was low and that the content of HMPA still remained high after the film was immersed in water for 5 min. In addition, in less than 5 min after immersion, phase separation of the casting solution occurred, evidenced by the casting solution turning opaque. The results indicate that as phase separation occurred, the solvent concentration at the film surface was still high (i.e., with a low polymer concentration), supporting the mechanism proposed by Bottino et al. [12].

With the relative solvent removal rates presented in Figure 10, we can satisfactorily explain the dependence of membrane permeabilities on solvent dipole moments, with HMPA, NMP or DMAc as the solvent. With a higher solvent dipole moment, the solvent removal rate in the coagulation bath was lower, indicating a higher solvent (lower polymer) concentration as phase separation occurred, reasonably explaining why the membrane permeability was higher. The above discussion supports that the solvent removal rate during membrane formation played an important role in determining the membrane permeability to water. However, the low water permeability of the membrane prepared with TEP as the solvent cannot be explained by considering the solvent removal rate alone. In addition to the solvent removal rate, the time period needed for phase separation to occur would also affect the polymer concentration as phase separation occurred. A longer time period would allow more solvent to be removed before phase separation occurred. The time period needed for phase separation to occur should be dependent on the nonsolvent (water) intake rate in the coagulation bath. Since TEP has a lower dipole moment than the other three solvents, the water intake rate should also be lower since it has less affinity for water. The lower water intake rate would allow more time for TEP to be removed before phase separation occurred, which could lower the solvent concentration at the surface of the cast film and result in a denser membrane surface. The lower affinity of TEP for water is believed to be the reason why the PVDF membrane prepared with TEP as the solvent had the lowest water permeability in Figure 8b, even though the removal rate of TEP was not the highest.

On the basis of the above discussion, it can be summarized that the monotonous increase in membrane permeability with the increasing solvent dipole moment can be reasoned by considering the solvent removal rate of the cast film in the coagulation bath and the effect of the solvent’s affinity for water.

We measured the retention of PVP (with a molecular weight of 55,000 g·mol^−1^) to characterize the separation performance of the prepared PVDF membranes. The results are presented in Table 3. For the membranes prepared with TEP, DMAc or HMPA as the solvent, their PVP retentions were higher than 90%, indicating the membranes’ MWCOs were smaller than 55 K. The PVDF membrane prepared with NMP as the solvent had a lower retention of the used PVP. The relationship between PVP retention and the dipole moments of the solvents is depicted in Figure 8c. By comparing the results presented in Figure 8b,c, it can be seen that the membranes with higher water permeability had lower PVP retentions, except for the membrane prepared with HMPA as the solvent. As discussed above, the high membrane permeability with HMPA can be explained by the high solvent concentration as phase separation occurred, resulting in high membrane porosity. The mechanism for the high membrane retention is still not clear. One possibility is that the high viscosity of the HMPA casting solution hindered the growth and coalescence of the polymer-poor domains formed after L-L demixing, resulting in smaller membrane pores. More investigations are still needed to clarify the underlying mechanism.

### 3.4. Mechanism Responsible for the Dependence of Solvent Removal Rates on Solvent Dipole Moments

As discussed in previous sections, the solvent removal rate from a cast film in the coagulation bath was a critical factor in determining the water permeability and the formation of crystalline phases in the prepared PVDF membrane. Moreover, as indicated in Figure 10, the solvent removal rates strongly depended on their dipole moments. The mechanism responsible for this dependence is discussed in this section.

**Figure 11:**
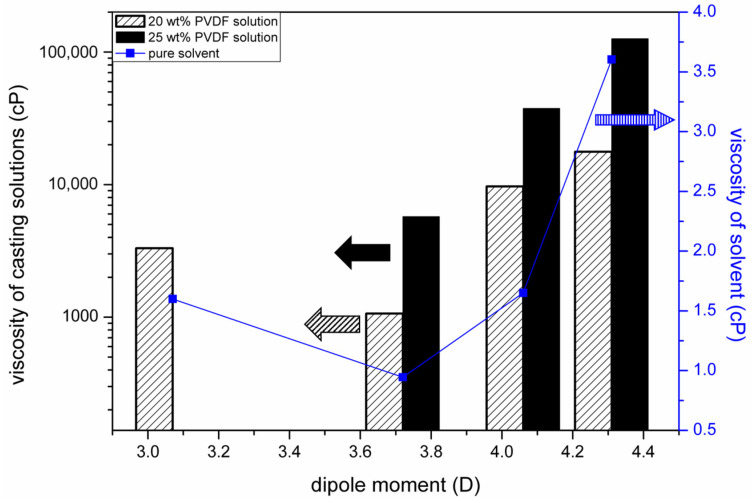
Dependence of the viscosities of solvents and casting solutions on solvent dipole moments.

The work of Bottino et al. [12] pointed out that membrane permeabilities were strongly related to the mutual diffusivities of the nonsolvent (water) and the solvents used to dissolve PVDF. They estimated the solvent–nonsolvent diffusivities by using the Wilke–Chang equation [59], in which the solvent viscosities play an important role in the estimation of diffusivities. We, therefore, plotted the dependence of the solvent viscosities on their dipole moments, as shown in Figure 11. In the same figure, we also plot the relationship between the viscosities of the casting solutions (20 wt% and 25 wt% of PVDF) and the solvent dipole moments. It can be seen that the dependence on the solvent dipole moments of the viscosities of the casting solutions has a similar trend as that of the solvent viscosities. The similar trends indicate that the viscosities of the PVDF solutions were strongly dependent on the interactions between solvent molecules. Since the solvent dipole moment is a measure of the interactions between the solvent molecules, a correlation between the viscosity of the casting solution and the solvent dipole moment was observed.

It can be deduced that the solvent removal rates were strongly related to the viscosities of the casting solutions, from the comparison of the results shown in Figure 10 and Figure 11. An increase in solution viscosity indicated a decrease in the solvent removal rate. The results reveal that the solvent removal rate from a casting solution was governed by the mass transport resistance in the casting solution. In other words, a high solution viscosity hindered the solvent diffusion in the solution and resulted in a low solvent removal rate. With the effect of the mechanism of viscous hindrance on solvent transport, we can reason the dependence of the solvent removal rate on its dipole moment.

## 4. Conclusions

1. Both the fraction of the polar crystalline phase (β) and the water permeability of the prepared membrane increased monotonously with the increasing solvent dipole moment.
2. The increase in membrane permeability with the increasing solvent dipole moment can be explained by the change in the solvent concentration at the surface of the cast film. A higher solvent concentration at the film surface led to a more porous surface with higher permeability.
3. The solvent content in the crystallization environment played a critical role in the formation of PVDF crystalline phases. A high solvent content allowed the solvent polarity to govern the formation of crystalline phases. With a low solvent content, the dominant crystalline phase was α, the kinetically favored one.
4. The solvent removal rate from a cast film strongly influenced the fraction of the β phase and the water permeability of the prepared PVDF membrane. The removal rate was dependent on the dipole moment of the solvent, and the dependence can be well explained by the change in the viscosity of the casting solution.
5. FTIR/ATR analyses were performed at the surfaces of the cast films at different time points during membrane formation to provide information on when crystallization occurred, how crystalline phases evolved, and whether or not the solvents were present in cast films as PVDF crystallized. The technique was shown to be a useful tool to obtain insight into the formation of membranes with semi-crystalline polymers.

## Figures and Tables

**Table 1:** Properties of the solvents used to dissolve PVDF.

Solvent	HMPA	NMP	DMAc	TEP
Dipole moment (D)	4.31 [36]	4.06 [37]	3.72 [38]	3.07 [39]
Viscosity (cP) at 25 °C	3.605 [40]	1.65 [41]	0.945 [42]	1.6 [43]
IR characteristic peaks (cm^−1^)	744, 992 [44]	1112, 1300 [45]	1011 [46]	966, 1021 [47]

**Table 2:** Characteristic IR and XRD peaks of α-, β- and γ-PVDF crystalline phases.

	FTIR	XRD
Crystalline Phase	Wavenumber (cm^−1^)	2θ (°)
α	760	18.30
	975	19.90
		26.56
β	840	20.26
	1275	41.20
γ	834	20.04
	1234	

**Table 3:** Properties and performance of membranes prepared with NIPS using various solvents.

Solvent	PVDF Concentration (wt%)	Degree of Crystallinity (%)	Ratio of β Phase (%)	Water Permeability (LMH/bar)	PVP Retention (%)
TEP	20	54.3	0	11.19	98.36
DMAc	20	51.6	28.3	12.10	90.39
DMAc	25	47.4	23.5	1.59	92.72
NMP	20	47.5	42.3	142.72	18.52
NMP	25	53.5	68.0	28.98	42.74
HMPA	20	58.6	100	246.47	91.28
HMPA	25	57.4	100	154.68	92.73

## Data Availability

The data presented in this study are available on request from the corresponding author.
